# Effects of auditory white noise stimulation on sustained attention and response time variability

**DOI:** 10.3389/fpsyg.2023.1301771

**Published:** 2023-12-08

**Authors:** Jens Egeland, Olaf Lund, Iwona Kowalik-Gran, Anne Kristine Aarlien, Göran B. W. Söderlund

**Affiliations:** ^1^Divison of Mental Health & Addiction, Vestold Hospital Trust, Tønsberg, Norway; ^2^Department of Psychology, University of Oslo, Oslo, Norway; ^3^Faculty of Teacher Education Arts and Sports, Western Norway University of Applied Sciences, Sogndal, Norway; ^4^Department of Education and Special Education, University of Gothenburg, Gothenburg, Sweden

**Keywords:** noise, sustained attention, Conners’ CPT-3, ADHD, arousal

## Abstract

**Introduction:**

“The moderate brain arousal model” claims that white noise improves attention by optimizing brain arousal. We analyze Conners’ Continuous Performance Test-3 (CCPT-3) performance, expecting to find reduced reaction time variability with noise mediated by decrease under long event-rates and in later parts of the test, indicating that noise reverse fall in phasic and tonic arousal.

**Methods:**

Sixty-five children with high or lower ADHD-symptoms from a child psychiatric unit, succeeded to complete the CCPT-3 with and without white noise.

**Results:**

Noise reduced overall variability, improved performance in later parts of the test, and reduced response variability under the longest event rate particularly in the high symptoms group. No overall change in omissions and commissions, but the high symptoms group made fewer omissions during noise compared the low symptom group.

**Discussion:**

The study indicates an arousal effect of noise but should be replicated with other noise variants and amplitudes to improve effect and compliance.

## Introduction

Several studies have shown that auditory “white noise” can extend attention span or improve working memory (Helps et al., 2014; Söderlund and Nilsson Jobs, 2016) and episodic learning (Söderlund et al., 2007, 2010) in children with attention deficits and/or ADHD. The same level of auditory stimulation will, however, lead to decreased performance in children with normal or good attention (Söderlund et al., 2007, 2010; Helps et al., 2014). Departing from the observation that all organisms perform best under optimal stimulation levels, “the moderate brain arousal model” (MBA model; Sikström and Söderlund, 2007) claims that white noise can increase the target stimulation level through the phenomenon of stochastic resonance, where weak signals can be reinforced by the presentation of random white noise together with the target signal. Apart from specifying the phenomenon of stochastic resonance, the predictions about white noise benefit are similar to the claim by arousal models of ADHD that the brain arousal necessary for successful task completion among children with ADHD may lead to over-arousal in typically developing children, resulting in lower performance (Sonuga-Barke et al., 2010; Martella et al., 2020).

Over the past decades, several researchers have suggested that ADHD is associated with a hypo-aroused brain state (Clarke et al., 2020), which causes compensatory motor hyperactivity and slow and variable response patterns observed on sustained attention tasks (Geissler et al., 2014). Hyperactivity is then considered to be an autoregulatory response to a hypo-aroused state (Hegerl and Hensch, 2014). Pribram and McGuinness (1975) were the first to distinguish between phasic and tonic arousal level and point to state regulation impairments as important to understand attention disorders. In ADHD, the tonic and phasic regulation of dopamine in the brain has been especially studied, as tonic levels of dopamine are assumed to be low (Volkow et al., 2009), and that low tonic DA leads to higher phasic release (Seeman and Madras, 2002) in accordance with the tonic-phasic model of dopamine signaling (Grace, 1991). The MBA model thus posits that people with ADHD are more dependent on externally evoked phasic dopamine to raise arousal to levels effective for performance (Ziegler et al., 2016). Central stimulating agents and white noise are both possible ways to increase phasic dopamine and thus increase performance under optimal stimulation and decrease performance when leading to over-arousal (Del Campo et al., 2011).

Behavioral correlates to these concepts were further developed in the cognitive energetic model (CEM) of ADHD (Sergeant, 2005), which, similarly to the MBA model, claims that children with ADHD have an insufficient regulation of brain states. In CEM (Sergeant, 2005), the phasic response to stimulation, influenced by signal intensity and novelty, is called the arousal pool, while the tonic level of arousal is influenced by the activity level of the subject and is called the activation pool (Martella et al., 2020). Varying event rates, i.e., stimulation level in Continuous Performance Tests (CCPTs), is a way to manipulate arousal, while the activity level during the task may exhaust or increase central nervous tonic energy resources (Martella et al., 2020). Thus, Loo et al. (2009) considered the quantitative EEG activation change from eyes closed to eyes open as a change in phasic arousal, while activity change due to responding in the continuous performance test was considered as activation mediated, i.e., measure tonic arousal. Loo et al. (2009) found that adults with ADHD required more cortical arousal in order to sit still in front of the computer and that successful performance on the test demanded more tonic arousal.

Loo et al. (2009), Zhou and Luo (2022), and Lin (2022) are three of the few researchers explicitly linking predictions from the MBA model and the CEM. Lin (2022) found that preschoolers with ADHD normalized their overall performance under white noise conditions on the Conners’ Kiddie Continuous Performance Test. To our knowledge, however, no study has actually tested whether noise benefits could be linked to presumed arousal or activation improvement in sustaining attention. Apart from the new Lin’s (2022) study, the beneficial effect of noise has previously been tested in relatively short intervals, for instance, for the duration of a short memory test with and without noise. More studies are needed to conclude a beneficial effect of working with concomitant noise over a longer period of time (i.e., 14 min), and we need to know whether a beneficial effect can be interpreted in the terms posited by activation models of ADHD, i.e., that improvement is due to normalization of their state regulation deficit during noise.

The most sensitive measure of sustaining attention in tests of sustained attention is reaction time variability or the standard deviation of the reaction time (Kofler et al., 2013; Salum et al., 2019; Pagán et al., 2022). Reaction time variability is particularly sensitive to ADHD by measuring occasional lapses in attention and/or failure to sustain attention that are not severe enough to result in omission errors (Huang-Pollock et al., 2012; Tamm et al., 2012). Reaction time variability is also shown to be most sensitive to central stimulating medication (Levy et al., 2018; Fredriksen et al., 2021). While the standard deviation of the RT is a gross measure of overall variation during the test, the Conners’ Continuous Performance test, version 3 (CCPT-3; Conners, 2014) also offers other measures of variability in performance. The test differentiates between three levels of stimulation intensity, i.e., stimuli might appear with *1, 2, or 4 s* of inter-stimulus intervals (ISIs). Performance under varying event rates may serve as an operationalization of phasic arousal effects in the sense that longer ISIs represent a less arousing environment, causing under-arousal in ADHD. A series of studies have found the ISI effect to be the most specific measure of ADHD-related inattention in CCPT (Egeland et al., 2009, 2012; Miranda et al., 2012; Udal et al., 2014; Chiang et al., 2015). Persons with ADHD are found to be performing less impaired in the high-intensive condition with one-second ISI’s, while persons with inattention due to other disorders such as schizophrenia typically perform best under low-intensive stimulation, i.e., 4-s ISI (Egeland, 2012; López-Luengo et al., 2016; Lundervold et al., 2016). The findings by Epstein et al. (2011) sum up the effects described above by finding no medication effect for the mean reaction time nor for omission errors, but that reaction time variability and particularly the coefficient of variation was sensitive to medication and incentives and specifically under low event rates (i.e., long ISI’s).

Another measure of intra-test variability is the block-change measure. It refers to the slope of change in reaction time as a function of time on task, and it serves as an operationalization of tonic arousal changes. Even though many subjects with ADHD have problems attending from the first part of the test, several studies have also found a decay in performance, satisfying the criteria for a specific deficit in sustained attention (Egeland and Kowalik-Gran, 2010; Zane et al., 2016).

As variability scores have emerged as the most sensitive measure of ADHD-related attention deficit, researchers have pointed out that they are not normally distributed. Lapses in attention contribute to long reaction times, but there will be few short reaction times deviating equivalently from the mean. Thus, analyses applying ex-Gaussian methods have become increasingly popular. The ex-Gaussian distribution is an estimated distribution combining a normal distribution and an exponential distribution (Luce, 1986; Heathcote et al., 1991), where *mu* and *sigma* represent the mean and the standard deviation of the normal distribution, respectively. *Tau* describes the mean and the standard deviation of the exponential distribution. This latter measure seems to be a more sensitive measure of RT variability, found to be higher in individuals with ADHD than in healthy controls (Bella-Fernández et al., 2023). Comparing subjects with ADHD and typically developing children, Gu et al. (2013) and Hwang-Gu et al. (2019) found deviating Tau-values both for longer ISIs and later time blocks in ADHD, summing up that their findings reflected an energetic problem in ADHD. If this is so, and noise reduces the energetic impairment, there should be a disproportional improvement by noise on ISI and block change. This is the topic of the present study.

In the present study, we analyze the effect of white noise in CCPT-3 (Conners, 2014). White noise is a random signal with a flat power spectrum having equal intensity over the entire frequency band. According to stochastic resonance, this would mean that white noise, as opposed to other types of noise, increases the neural signal-to-noise ratio equally in the entire spectrum of frequencies that human ears can encode. The white noise signal is considered to interact with the target signal in the narrow time window when neurons are close to the threshold of eliciting an action potential. The noise thus pushes the signal over the threshold, i.e., resonance.

We have applied the same high level of noise usually applied in studies of clinical samples, although most of these studies deliver the noise for shorter periods of time. One study of neurotypical adults suggested that the same level of noise that was optimal for working memory tasks was too high for sustained attention (Awada et al., 2022). This study, however, was performed on neurotypical adults, where we generally do not expect the positive effects of noise.

Summing up, the MBA model of Sikström and Söderlund (2007) resembles the CEM of ADHD (Martella et al., 2020) by emphasizing the value of optimal arousal level. White noise is found to increase performance in many cognitive tasks, and we expect it to also improve sustained attention. A group of drug-naive children, referred for neuropsychological assessment of attention deficits, is tested with CCPT-3 two times: with or without white noise. We expect (1) better performance under white noise conditions evident in a reduction of variability of response time, i.e., smaller standard deviations of reaction times and a smaller between-block variability. (2) We further expect that the improvement is related to arousal, i.e., that the improvement will be specific to the low-intensity stimulation part of the test and the latter part of the test. We divide participants into those having high or low ADHD symptoms. We have no hypothesis of significant group differences as both groups display attention problems, but we expect that the ISI and block effects will be mainly driven by those with higher scores of ADHD symptoms.

## Methods

### Participants

Children successively referred for neuropsychological assessment in the Child Psychiatric Clinic of Vestfold Hospital Trust, Norway, were invited to take part in the study. The children were referred on the suspicion of problems with attention, executive function, tics, specific learning disorders or dyslexia. A total of 83 children accepted to take part. If the computer scoring program for CCPT-3 did not yield a measure of variability, the test was not considered to be valid. This means that the participants had to have at least three responses to stimuli in each of the 18 sub-parts of the test. In total, four of the subjects did not perform the CCPT validly without noise (three with no noise as the first condition), 10 subjects declined to start the session with noise, and eight subjects starting the session with noise had periods without responding, resulting in no variability measure computed. They were, therefore, considered as drop-outs (all eight had noise as the second condition). In total, 65 participants performed both test sessions validly. Whether starting with noise or without was randomized, but in the final sample of 65 completers, 53% had started without noise and 47% with noise.

The project was approved by the Regional Ethics Committee (2018/446–3) and by the Data Protection Officer of Vestfold Hospital Trust. The data are stored on a secure research server of Vestfold Hospital Trust but can be made available on demand by e-mail to forskning@siv.no. The research was funded by the Norwegian Research Network on ADHD, grant number 18/09147–5.

Of note, 36 boys and 29 girls participated. The mean age was 11.9 years, ranging from 8 to 18 years of age (SD = 2.9).

All data, except for the CCPT-3, were taken from patient file records. Patients were examined with somewhat different scales of attention and psychiatric symptoms. Participants with a T-score above 65 on the Child Behavior Checklist (Achenbach, 1991) DSM ADHD scale were considered to have significant ADHD symptoms. A total of 38 participants scored above that level, while 20 scored below, and CBCL were lacking for seven participants due to parents not being able to fill out a Norwegian questionnaire or due to technical errors (failure in scanning the correct pages into patient files). For four of those lacking CBCL, we used the comparable Achenbach’s Teacher Report Form (TRF) filled out by their teachers (Achenbach, 1991). The remaining three were classified into significant ADHD scores or not based on their ADHD Rating Scale, version 4 (ARS-4; DuPaul et al., 1998) scores.

Table 1 shows clinical data differentiating those classified as below or above the cut-off for significant ADHD symptoms. On CBCL, the two groups differed with regard to symptoms of ADHD, ODD, and CD. On TRF, they differed only with regard to the level of ADHD symptoms. On Behavior Rating Inventory of Executive Function (BRIEF), they differed with regard to inhibition but not with regard to working memory, indicating an impairment in both the ADHD group and the non-ADHD group consisting of clinical cases with other diagnoses expected to also score high on WM. In the same vein, ARS showed no difference in the Inattentive scale but a significant difference regarding the hyperactivity/impulsivity scale. In the forthcoming analyses on CCPT-3 performance, raw scores will be analyzed, but for the information of the reader, the T-scores of the two subgroups are reported here. The two groups differed with regard to the number of omissions, the standard deviations of hit reaction time, and variability. The sample did not differ with regard to ISI change.

**Table 1:** Clinical data describing those classified below or above the cut-off for significant ADHD symptoms (*N* = 65).

	Below ADHD cut-off (*N* = 23)	Above ADHD cut-off (*N* = 42)	F	p
Age	12.2 (3.0)	11.8 (2.8)		
Gender (G/B)	7/15	22/20		
**CBCL—DSM-scales (** ***N*** **= 58)** ^**1**^	*N* = 20	*N* = 38		
Affective disorders	66.7 (8.8)	69.6 (8.0)	1.7	n.s.
Anxiety	70.0 (14.2)	69.2 (11.3)	0.6	n.s.
Somatic disorder	623 (9.2)	62.0 (9.7)	0.013	n.s.
ADHD	58.0 (3.7)	72.7 (7.1)	73.6	< 0.001
ODD	61.9 (7.3)	66.7 (8.1)	4.8	0.033
CD	60.9 (8.2)	70.4 (8.8)	16.2	< 0.001
**TRF—DSM-scales(** ***N*** **= 41)** ^**2**^	*N* = 11	*N* = 30		
Affective disorders	62.3 (6.1)	65.3 (8.2)	1.2	n.s.
Anxiety	64.4 (13.8)	62.4 (10.5)	0.2	n.s.
Somatic disorder	55.6 (10.6)	59 (11.3)	0.8	n.s
ADHD	58.0 (5.1)	68.3 (13.4)	6.1	0.018
ODD	57.8 (8.3)	63.3 (8.3)	3.5	n.s.
CD	58.3 (7.5)	64.9 (11.9)	2.9	n.s.
**BRIEF (** ***N*** ** = 54)** ^**3**^	*N* = 17	*N* = 37		
Inhibition	56.5 (12.1)	70.0 (14.5)	11.1	0.002
Working Memory	69.0 (10.6)	72.4 (10.1)	1.3	n.s.
BRI^4^	63.6 (10.9)	73.2 (12.5)	7.3	0.009
MI^5^	65.8 (15.1)	71.5 (9.2)	4.2	0.045
**ARS** ^**6**^ **(** ***N*** ** = 39)**	*N* = 12	*N* = 27		
Inattention	16.3 (5.0)	18.3 (5.7)	1.1	n.s.
Hyperactivity/Impulsivity	7.4 (5.4)	15.3 (7.7)	11.8	0.001
**CCPT-3 without noise** Omissions *t* -score (*n* = 65)	53.5 (10.8)	62.2 (15.6)	5.6	0.021
Hit RT s.d.	53.5 (11.4)	63.8 (14.6)	8.5	0.005
Variability	52.3 (11.6)	59.7 (11.6)	6.0	0.017
ISI Change	53.2 (9.5)	58.1 (12.8)	2.5	n.s.
Block Change	50.8 (10.4)	54.7 (13.3)	1.5	n.s.
Full scale IQ	92.0 (11.9)	91.1 (13.6)	0.6	n.s.

^1^Child Behavior Checklist; ^2^Teacher Report Form; ^3^Behavior Rating Inventory of Executive Function, parent version; ^4^Behavior Regulation Index; ^5^Metacognition Index; ^6^ADHD Rating Scale.

### Instruments

#### Conners’ continuous performance test, version 3

All participants were tested with CCPT-3 (Conners, 2014) two times, with or without concomitant white noise. In the CCPT-3, the participant is exposed to 360 sequential exposures of letters on a computer screen and is asked to respond as quickly as possible to all letters except X’es, i.e., to 80% of all exposures. After qualifying in a 1-min training session, the entire test lasts for an additional 14 min, with letters being exposed to three different ISIs of 1, 2, or 4 s. For computing changes over time, the test is divided into six time blocks, which then are further subdivided into blocks varying in the three different ISIs, altogether 18 sub-blocks.

A number of omission errors, the standard deviation of the reaction time (hit RT s.d.) and variability are considered most sensitive to overall attention in the test (Egeland and Kowalik-Gran, 2010). While the hit RT s.d. is computed from all correct responses to target items, the variability score is computed as the variation in reaction time between the 18 sub-blocks. Commission errors are the number of false-positive responses to X’es. Block change is a regression factor showing the slope of change in reaction time as time on task. The ISI change is the slope of change in reaction time over the three ISIs. All variability measures are computed without logarithmic transformation.

Since we are particularly interested in the ISI effect, we compare noise-related changes in omission errors to split up into ISI conditions. We report all three ex-Gaussian measures but are expecting only the Tau value to be related to noise effects. This measure is split up into ISI conditions to check for differences in the exponential curve of reaction time by ISI and noise. Due to data loss, the ex-Gaussian measures were computed from 64 rather than 65 persons.

#### Rating scales

Parents and teachers completed the respective Achenbach scales, namely the Child Behavior Checklist (CBCL) for parents and the Teacher Report Scale for teachers (Achenbach, 1991). These checklists provide scores for different child psychiatric symptoms as well as DSM-diagnosis-oriented scales for ADHD. The Norwegian version has shown good reliability and validity (Nøvik, 2000). We also report scores for the ARS-4 (DuPaul et al., 1998) and from the Behavior Rating Inventory of Executive Function (BRIEF) parent version (Fallmyr and Egeland, 2011). This inventory gives scores for nine scales related to executive function in daily life, where high scores on impulsivity and working memory are typical for ADHD. Four scales measuring aspects of behavior regulation (including impulsivity) are merged into a composite index for behavior regulation, while the remaining five scales give a metacognition index score (among them, working memory). See Table 1, for figures.

### Procedure

The noise was delivered binaurally through high-quality headphones (Sennheiser model 201). After testing the noise set to 80 dB, we allowed for lowering the noise to 70 dB if the children preferred so. White noise in the present study was a 15-min recording from an old TV set that was not receiving any signal; this noise was then looped and run on the test computer. Most participants were randomized into either noise or no-noise first, but in some cases, the CCPT-3 had been performed as part of an initial clinical examination prior to joining the study, and a decision was made not to repeat the no-noise condition. The two CPT sessions were part of a more extended assessment, including WISC-V and test screening for language impairments. In all together 21 cases, a decision was made to divide the testing into two sessions on 2 days. For 44 participants, both CCPT test sessions were performed on the same day, but the children were offered a 15-min break before starting session 2. All participants were tested individually in a silent room alone together with qualified staff personnel (coauthors I.K.G and AKAa).

### Statistical analyses

Data were analyzed with paired-sample *t*-tests comparing noise and no-noise conditions. Since we had an expectation of improvement by white noise, a one-tailed *t*-test with an alpha level of <0.05 was applied. Ex-Gaussian parameters were estimated from the correct responses to target items for each participant, using R Statistical Software (v4.2.1; R Core Team, 2022) and the R Package retimes (Massidda, 2013). Parameters for Mu, Sigma, and Tau are reported for the whole test, and we also report parameters for Tau from each ISI condition. Power analyses showed that a sample of 65 would yield 80% power to detect small effects exceeding Cohen’s *d* of 0.26. We had no firm hypothesis of significant group differences but expected the group with high ADHD symptoms to improve somewhat more from noise. Thus, the data file was split into low- and high-ADHD symptom scores and the analyses were repeated in each of these subgroups.

## Results

Since we analyzed variability measures that were not log-transformed, we checked the kurtosis and skewness of these parameters. Skewness varied from 0.78 for variability in the no-noise condition to 1.49 for the ISI change in the noise condition. Kurtosis varied from −0.30 for variability in the no-noise condition to 3.06 for block change in the no-noise condition. The latter value indicates a small variation.

Table 2 shows the performance of the whole group split up with regard to low or high ADHD symptoms. For the whole sample, there was a positive effect of noise for all variability measures (i.e., the standard deviation of the reaction time, block change and ISI change and variability). There were no differences between the number of omissions and commissions, but splitting up into ISIs, there was a decrease in omissions under noise conditions for the one-second ISI. Analyzing the ex-Gaussian measures showed an overall effect of reduced Tau due to noise but not with regard to Mu and Sigma. Splitting up into ISI showed that the noise effect on Tau was driven by the significant reduction of response time variability in the 4-s ISI condition.

**Table 2:** CCPT-3 performance without and with white noise for the whole sample and split with regard to low−/high-ADHD symptoms.

	Whole sample *n* = 65					CBCL ADHD<t65 *N* = 23					CBCL ADHD > t65 *n* = 42				
	No noise M (S.D.)	Noise M (S.D)	t	p	Cohens d’	No noise M (S.D.)	Noise M (S.D.)	t	p	Cohens d’	No noise M (S.D.)	Noise M S.D	t	p	Cohens d’
Hit Rt s.d.	0.218 (0.129)	0.194 (0.110)	2.17	0.019	0.21	172 (95)	161 (102)	0.826	n.s.	0.11	243 (123)	213 (111)	1.95	0.029	0.26
Block change	10.78 (19)	6.77 (15)	1.68	0.049	0.23	5.70 (14.9)	3.37 (11.30)	0.55	n.s.	0.18	13.6 (20.7)	8.6 (16.6)	1.80	0.049	0.26
ISI change	0.45 (0.32)	0.39 (0.29)	1.75	0.040	0.19	36 (24)	31 (28)	0.961	n.s.	0.18	49 (35)	42 (29)	1.48	n.s.	0.19
% Omiss.	7.18 (7.0)	6.21 (5.85)	1.58	0.059	0.15	4.67 (4.73)	5.37 (5.79)	−0.919	n.s.	−0.13	8.55 (7.74)	6.67 (5.90)	2.38	0.011	0.26
% omiss. ISI 1 s.	8.26 (8.75)	6.47 (6.47)	1.86	0.034	0.22	5.91 (6.38)	6.84 (8.19)	−0.598	n.s.	−12	9.49 (9.65)	6.27 (5.41)	2.82	0.004	0.36
% omiss. ISI 2 s.	7.09 (8.11)	6.32 (6.52)	0.899	n.s.	0.09	4.45 (5.37)	5.03 (5.90)	−0.605	n.s.	−0.10	8.54 (9.01)	7.11 (6.80)	1.29	n.s	0.10
% omiss. ISI 4 s.	6.20 (6.73)	5.78 (6.99)	0.716	n.s.	0.06	3.60 (4.38)	4.26 (5.49)	−0.703	n.s.	0.13	7.62 (7.39)	6.62 (7.63)	1.36	n.s.	0.13
Commissions	53.71 (20.09)	52.82 (21.47)	0.587	n.s.	0.04	49.38 (20.45)	51.09 (19.74)	−0.680	n.s.	−0.08	56,08 (19.74)	53.77 (21.70)	1.23	n.s.	0.11
Variability	104 (77)	89 (69)	1.71	0.046	0.20	78 (69)	61 (51)	1.29	n.s.	0.27	118 (78)	104 (73)	1.19	n.s.	0.13
Rt Tau	162 (91)	145 (86)	1.87	0.041	0.22	122 (62)	131 (91)	−0.670	n.s.	−0.14	184 (96)	155 (81)	2.58	0.009	0.40
Rt Mu	319 (57)	315 (56)	0.672	n.s.	0.07	337 (36)	322 (52)	1.64	0.058	0.35	310 (63)	312 (59)	−0.229	n.s	−0.03
Rt Sigma	71 (31)	68 (32)	0.966	n.s.	0.09	65 (28)	58 (29)	1.92	0.034	0.41	73 (32)	73 (32)	−0.150	n.s.	−0.23
ISI1 Tau	105 (46)	101 (54)	0.597	n.s.	0.07	115 (60)	130 (96)	0.808	n.s.	0.037	115 (47)	100 (58)	0.1.84	0.037	0.28
ISI2 Tau	149 (85)	145 (93)	0.434	n.s.	0.04	86 (38)	104 (47)	3.96	n.s.	0.158	167 (91)	152 (92)	1.07	n.s.	0.17
ISI4 Tau	196 (128)	163 (107)	2.86	0.003	0.35	145 (89)	140 (110)	0.132	n.s.	0.006	222 (138)	175 (104)	3.19	0.001	0.49

Splitting up into low and high ADHD symptoms showed reduced standard deviation of the reaction time, improved block change, and reduced number of omissions in the ISI 1-s condition in the noise condition only in the group with high ADHD symptoms. While there was no overall reduction in omissions due to noise, the splitting into two groups showed that the high-ADHD-symptom group had a significant reduction in omissions, while the low-ADHD-symptom group had a numeric increase in omissions. This group × noise interaction was significant (*F* = 4.32, *p* = 0.042, Eta^2^ = 0.064).

The overall differences in Tau and specifically in the ISI 4-s condition were driven by the high-ADHD-symptom group, showing significantly reduced variance in the noise condition.

## Discussion

The study aimed to test whether white noise could have a beneficent arousing effect on performance in a continuous performance test of attention. We had two sets of explicit hypotheses and one additional expectation. First, we hypothesized that overall variability would be reduced by white noise. This was confirmed by the finding that both the standard deviation of the reaction time and time-block variability were improved under noise conditions. This is in line with previous research showing that variability measures are more sensitive to inattention than manifest errors of omissions and commissions (Huang-Pollock et al., 2012). Analyzing the total sample, there were no changes in overall omissions and commission errors, but a significant reduction by noise was found in the subgroup with high ADHD symptoms.

Our second hypothesis was that the overall improvement by noise would be mediated by changes, especially under long event rates and later time blocks. Event rates represent different short-term changes in the intensity of external stimulation, considered to be a proxy of changes in phasic dopaminergic neuromodulation, previously found to be improved by white noise (Rausch et al., 2014). The fall under the no-noise condition in sustaining performance over time is related to a fall in tonic arousal level, as shown by Shalev and Nobre (2022), taking long-scale pupil dynamics to measure tonic arousal. The hypothesis was confirmed in the sense that white noise improved performance under the longest event rate but not under the shortest and the intermediate ISI and that the block-change measure showed an increase in performance due to changes in the later parts of the test. Thus, the improvement reduced the low arousal deficit characteristic of ADHD according to the CEM (Hwang-Gu et al., 2019) and the MBA model (Sikström and Söderlund, 2007; Söderlund et al., 2016).

We had no hypothesis regarding significant differences between the high- and low-ADHD-symptom groups, as both groups included clinical subjects referred for assessment of potential attention deficits. However, we expected the arousal-related performance effect to be mostly driven by those with high ADHD symptoms, and this was confirmed in that the effect sizes were numerically higher in this group. In fact, we also found a double dissociation with regard to omission errors, as they improved in the high-ADHD symptom group but decayed under noise conditions in the low-ADHD symptom group.

One finding that did not fit the expectations is the finding that the subjects with high-ADHD symptoms had fewer omissions in the 1-s ISI and that Tau showed less variability of reaction time also under the 1-s ISI. The effect size of the latter finding was, however, clearly lower than the effect under 4-s ISI. The potential for a reduction in omissions was larger in the 1-s ISI, but we acknowledge that it is not as expected.

The effect sizes of noise were mostly small to medium. It is also important to stress that the noise condition was unpleasant for several children, in the sense that some children refused to take the test or quit during the noise condition. Thus, we are hesitant to conclude that the noise effects shown in this study could be regarded as an effective tool for improving the attention of those with high ADHD symptoms.

On the other hand, this is the first study to find a significant association between noise benefit and a model for measuring arousal in attention. Several previous studies have shown a beneficial effect of noise for a short period but have not explicitly tested the brain arousal theory lying behind the noise benefit model of Sikström and Söderlund (2007). Loewen and Suedfeld (1992) suggested that auditory masking of naturalistic background noise could be one benign effect of noise, but the present study was performed in a silent room with no such possible distracting noise that could represent this alternative explanation of the noise effect.

Finding a small general regulating effect (the reduced reaction time variability) and the theoretically interesting ISI and block effects are encouraging and should lead to further research trying to improve the noise effect so that it could serve as a possible treatment. Since this study was started, both “pink” and “brown” noises have been suggested to have effects on cognitive functioning (Guo et al., 2022). White noise is a random signal with a flat power spectrum having equal intensity over the entire frequency band. To the participant, it sounds like a radio tuned to an unused frequency. Brown noise has a larger amount of lower frequencies than white noise, which does not have a flat power spectrum. It is a duller sound that resembles the sound of a river current or strong wind. Pink noise sounds like a waterfall. Considering the lack of studies, especially with subjects with ADHD, one nevertheless could speculate that the tolerability of the intervention would have been better if we had used pink noise. However, the effectiveness of the intervention might have been somewhat smaller for those who completed the trial with noise. We had, however, no possibility to test out different noise types and could neither test out different noise amplitudes. In the present study, it was 70–80 decibels, which could be too high for some individuals or too long period for some. A study by Awada et al. (2022) indicated that lower amplitude is best for sustained attention, while higher amplitude may be better for shorter working memory tasks in neurotypical young adults. An interesting idea is to let participants choose their own noise levels, thus using noise level as a dependent variable and thereby be able to explore if the preferred noise levels correlate with symptom severity in ADHD. Altogether, these results highlight the need for more research on noise levels, noise characters, and how to adapt these both to individuals and to different tasks. There is still a big gap in the literature before we can establish patient-specific guidelines for the use of noise stimulation or a best practice protocol for the administration of noise treatment (Pickens et al., 2019).

There are several limitations to the study. As it was integrated into clinical practice, we had to make some choices balancing between clinical demands and the rigor necessary for good research. While the majority were tested two times on the same day, some subjects were tested on different days. We have run all analyses limited to those tested on the same day, and it did not change any of the significant findings. We apologize that there are missing data points in the descriptive data, but for the most part, this is due to questionnaires only available in Norwegian, prohibiting many immigrant parents from filling them out. There were also examples of questionnaires belonging to the research protocol that were not applied due to the judgment of the treating clinician as not necessary. There is, however, no reason to believe that these inaccuracies will change anything regarding data.

However, a possible more important limitation is that the researchers administering the tests were not blinded as to the noise/no-noise conditions. Could it be that they unconsciously signaled an anticipation of better performance with noise? In fact, we consider it the other way around; the clinicians were faced with many negative comments from the participating children toward the high noise, and mid-way in the project, the administrators clearly signaled to the research group an expectation that noise would show up to be detrimental. In hindsight, it might be that these expectations lowered the threshold for letting participants abandon the repeated testing, particularly with noise.

Although we classify the participants into high or low levels of ADHD symptoms, they constitute a clinical sample; thus, we did not compare the two subgroups. If we had compared the high symptoms group to a neurotypical control sample, we would have expected significant differences. It follows that the benign effect of white noise in this study cannot be generalized to children in general. Since we not only find a benign effect of noise but also that its effect is driven by improved attention under long event rates and the last part of the test, the study should be replicated with a healthy control group to test the specificity of these mechanisms of change. We expect that the inverted U-curve of optimal stimulation is generally moved rightward among subjects with ADHD. However, in this study, we merely study half of the inverted U. We study contingencies varying from intense stimulation not in need of additional extrinsic stimulation (i.e., white noise) to low-intensity stimulation needing noise to raise attention, but we do not continue into an expected decay even when noise is added.

The participants are compared on 15 measures from the CCPT-3. A possible critique would be that we should have applied a Bonferroni correction for multiple comparisons. However, the analyses are theory driven rather than an exploratory search for significant results in one direction or the other. In fact, some of the noise/no-noise comparisons are performed just to be comprehensive with an explicit expectation of a null finding, such as the Mu and Sigma analyses and the Tau analyses of 1- and 2-s ISI. One could have analyzed only Tau 4-s ISI, but then the reader could question whether the finding was specific to the longest inter-stimulus interval or a general effect. Thus, many comparisons are necessary to give a detailed and nuanced picture of the relationship between noise and arousal.

In conclusion, trying to integrate the cognitive energetics model and the MBA by their joint focus on state regulation deficits in ADHD, we found indications that noise reduces what is considered indicative of arousal impairment, both by reducing reaction time variability and specifically by reducing the fall in attention under low-intensity-stimulus conditions and in later time blocks. The effect is small but encouraging and should lead to further research on varying noise conditions, in particular, while there is a demand for non-pharmacological treatment alternatives in ADHD.

## Data availability statement

The raw data supporting the conclusions of this article will be made available by the authors, without undue reservation.

## Ethics statement

The studies involving humans were approved by Regional Ethics Committee Sør-Øst. The studies were conducted in accordance with the local legislation and institutional requirements. Written informed consent for participation in this study was provided by the participants’ legal guardians/next of kin.

## Author contributions

JE: Conceptualization, Data curation, Formal analysis, Investigation, Methodology, Project administration, Software, Validation, Writing – original draft, Writing – review & editing. OL: Data curation, Investigation, Methodology, Software, Supervision, Writing – review & editing. IK-G: Data curation, Investigation, Methodology, Writing – review & editing. AA: Data curation, Investigation, Methodology, Writing – review & editing. GS: Conceptualization, Formal analysis, Methodology, Supervision, Writing – review & editing.
